# Supplementary material for: Campylobacter vaccination reduces diarrheal disease and infant growth stunting among rhesus macaques
Source: Nat Commun. 2023 Jun 26;14:3806. doi: 10.1038/s41467-023-39433-1 (PMC10293212; doi:10.1038/s41467-023-39433-1)
Supplement: Supplementary file 1 — Supplementary Information [file 41467_2023_39433_MOESM1_ESM.pdf]

Campylobacter vaccination reduces diarrheal disease and infant growth stunting among rhesus macaques

SUPPLEMENTARY INFORMATION

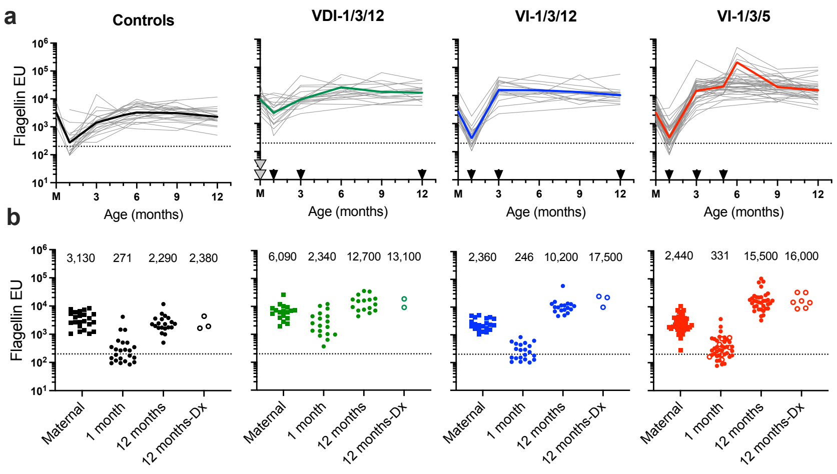

Supplementary Fig. 1. Comparison of *C. coli* Flagellin-specific serum antibodies elicited after natural infection or vaccination.
To measure antibacterial IgG responses against *C. coli*, serum samples were drawn at regular intervals from Control infants (1, 3, 6, 9, and 12 months), VDI-1/3/12 infants (1, 3, and 12 months), VI-1/3/12 infants (1, 3, and 12 months), and VI-1/3/5 infants (1, 3, 5, and 12 months, with a subset of 7 animals screened at 6 months). (a) Among animals with no history of *C. coli* diarrhea, *C. coli* ELISA Units (EU) were measured longitudinally for each infant (thin lines) and the geometric mean was calculated at each time point (thick lines). The 0 month time point represents maternal (M) antibody titers from serum samples of each infant's dam that were drawn at the infant's 1 month time point to estimate adult/maternal antibody levels in each cohort. Control infants were not vaccinated whereas the other three groups of infants were vaccinated on a 1, 3, 12 month schedule or a 1, 3, 5 month schedule, as indicated by the black arrow heads on the X axis. The gray arrow heads indicate that the dams in VDI-1/3/12 cohort were vaccinated twice during pregnancy. (b) Comparisons were made between the 1 month and 12 month time points among non-diarrheal animals within each group in addition to a comparison between 12 month time points from animals that were asymptomatically infected and/or vaccinated against *C. coli* (12 months) with infants from each group that were hospitalized for *C. coli* diarrhea (Dx) prior to the 12 month time point (12 months-Dx). No infants were diagnosed with diarrhea at 1 month of age but the open symbols at the 1 month time point represent animals that eventually were diagnosed with diarrhea by the 12 month time point. The numbers above each group represent the geometric mean antibody titer. Source data are provided as a Source Data file.

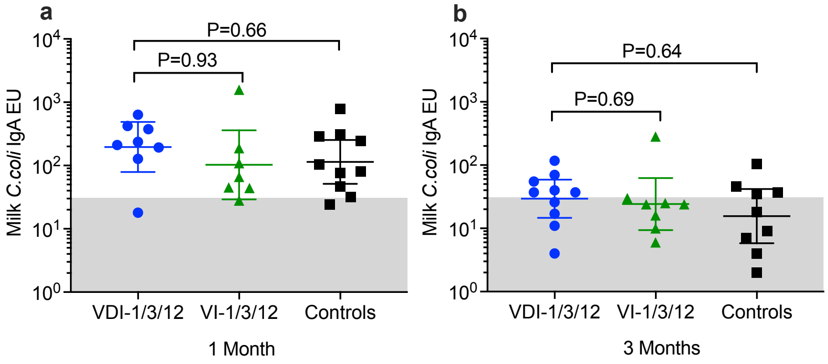

Supplementary Fig 2. Analysis of Campylobacter-specific IgA levels in Rhesus macaque breastmilk. In these studies, milk was expressed from lactating female RM at 1 month (a) and 3 months (b) after delivery in the VDI-1/3/12 (*n* = 8 at 1 month and *n* = 10 at 3 months), VI-1/3/12 (*n* = 7 at 1 month and *n* = 8 at 3 months), and Control groups (*n* = 10 at 1 month and *n* = 9 at 3 months) with the geometric mean antibody titers (± 95% confidence intervals) shown for each group. Milk sample volumes were limited and the highest concentration of milk was tested at a 1:30 dilution (the limit of quantitation is indicated by gray shading) and ELISA scores below 1:30 were extrapolated. Statistical comparisons were performed by One-way ANOVA with unadjusted P values. Source data are provided as a Source Data file.

EU; ELISA units

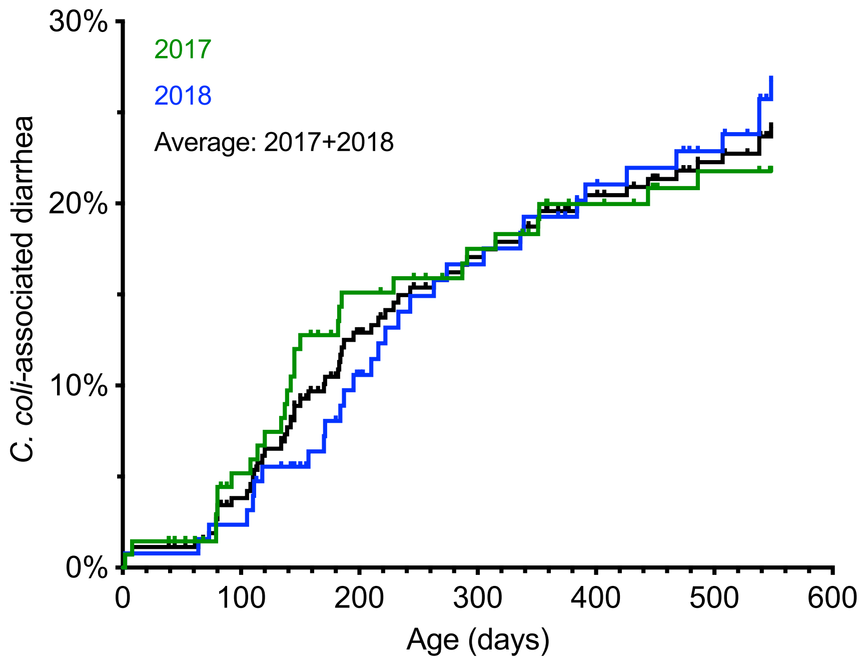

Supplementary Fig. 3. Comparison of *C. coli*-associated diarrhea incidence in 2017 and 2018.
In these studies, the age-associated incidence of hospitalization due to *C. coli*-associated diarrhea among infant rhesus macaques across the 2017 (n = 139) and 2018 (n = 127) birth cohorts was compared. Overall, the rates of *C. coli*-associated diarrhea were comparable in both years and resulted in approximately 20% hospitalization by 1 year of age. Source data are provided as a Source Data file.

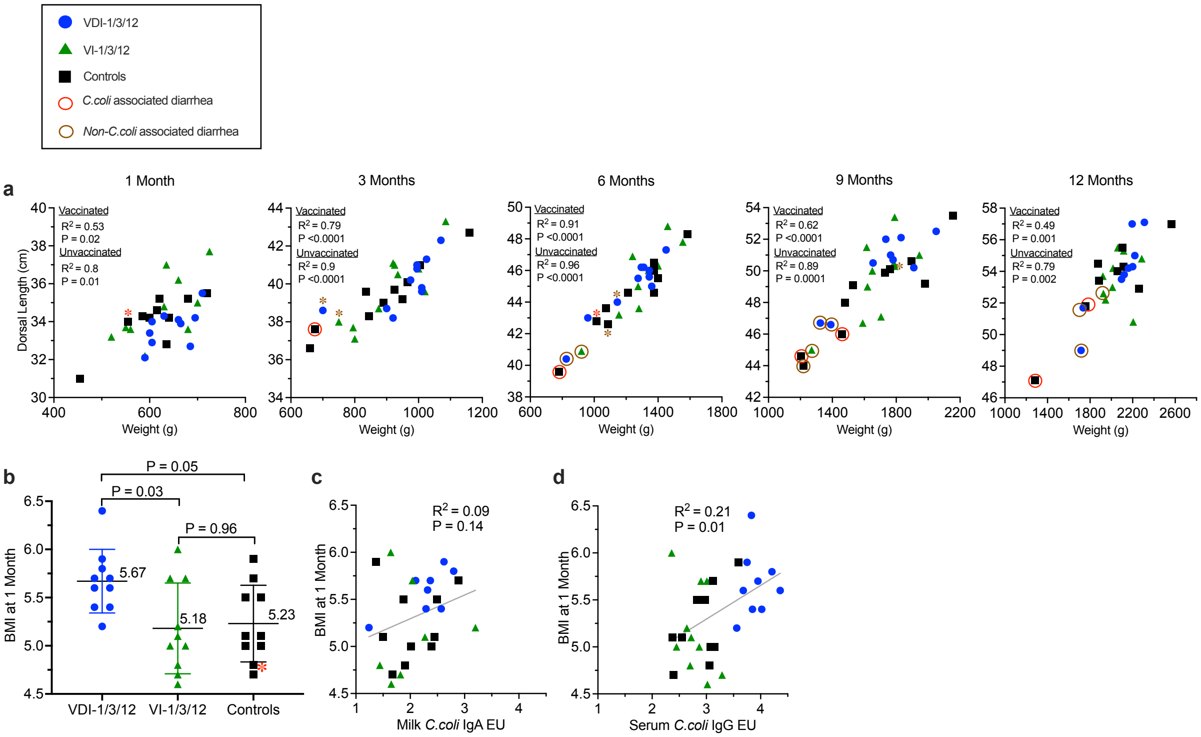

Supplementary Fig. 4. Analysis of weight gain among infant rhesus macaques.

Infant weight gain was measured longitudinally among a subset of Vaccinated infants and unvaccinated Controls. (a) At the indicated time points (1, 3, 6, 9, and 12 months), the individual weight (grams; g) and length (centimeters; cm) measurements for each infant macaque were graphed. At each time point, there was a significant correlation between infant weight and length. Symbols with an asterisk (*) indicate animals that appeared healthy at the time of measurement but were hospitalized with diarrhea at the next following time point. The study began with 10 vaccinated infants/group (*n* = 10 VDI-1/3/12 infants with 9 remaining at both 9 and 12 months and *n* = 10 VI-1/3/12 infants with 9 remaining at 12 months) and 10 unvaccinated Controls (*n* = 10 Control infants with 9 remaining at 12 months). Brown asterisks indicate future hospitalization with all-cause diarrhea and red asterisks indicate future hospitalization with *C. coli* diarrhea. Notably, animals hospitalized with diarrhea (indicated by circles) were disproportionally smaller in terms of both height and weight compared to their healthy/non-diarrheal counterparts. Brown circles indicate animals diagnosed with all-cause diarrhea whereas red circles indicate animals that were diagnosed with *C. coli*-associated diarrhea. Two-sided unadjusted P values were determined by univariable linear regression. (b) The 1 month of age data in panel A suggested that infants in the VDI-1/3/12 group (blue circles) were heavier for their length compared to the other infants. This was particularly interesting because all infants are exclusively breast-fed at this age. These potential differences were examined more closely by determining the body mass index (BMI) of each group at 1 month of age. The BMI of the VDI-1/3/12 infants (BMI = 5.67, *n* = 10) was significantly higher than that observed among VI-1/3/12 infants (BMI = 5.18, ANOVA post-hoc two-sided Tukey-adjusted P =0.03, *n* = 10) and unvaccinated Controls (BMI = 5.23, ANOVA post-hoc two-sided Tukey-adjusted P =0.05, *n* = 10) whereas there was no significant difference between VI-1/3/12 infants and unvaccinated Controls (ANOVA post-hoc two-sided Tukey-adjusted P =0.96). The mean BMI (± standard deviation) are shown for each group. Log-transformed *Campylobacter*-specific IgA in breastmilk (c) or *Campylobacter*-specific IgG in infant serum (d) were compared to infant BMI at 1 month of age. Two-sided unadjusted P values were determined by univariable linear regression and this data shows that although there was no correlation between breastmilk IgA titers and infant BMI (P=0.14), there was a significant correlation between infant serum IgG levels and improved infant BMI (P=0.01). Together, these results indicate that vaccinating pregnant dams resulted in higher BMI among their offspring at 1 month of age compared to infants born to unvaccinated dams and that this appears to be associated with *Campylobacter*-specific serum IgG titers. Source data are provided as a Source Data file.

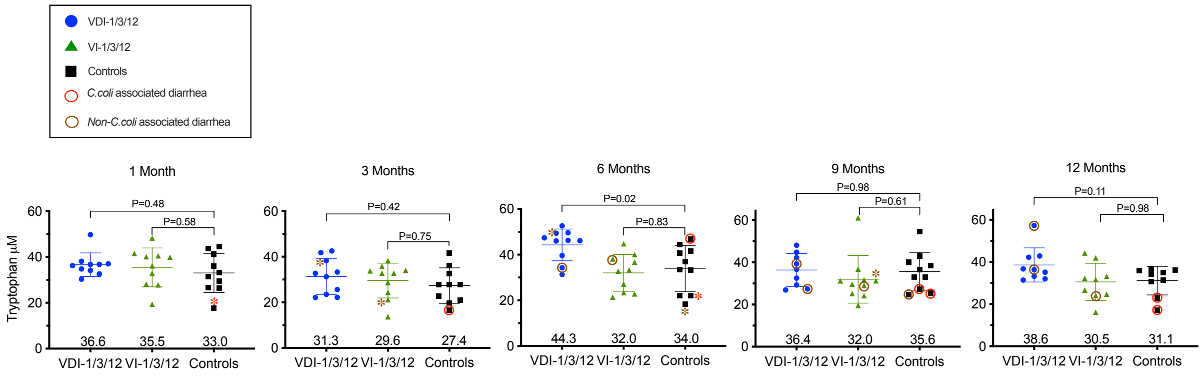

Supplementary Fig. 5. Comparison of serum tryptophan levels by vaccine group and age.
Serum tryptophan levels were measured longitudinally among a subset of Vaccinated infants and unvaccinated Controls. The mean tryptophan levels (± standard deviation) are shown for each group. Symbols with an asterisk (*) indicate animals that appeared healthy at the time of measurement but were hospitalized with diarrhea at the next following time point. The study began with 10 vaccinated infants/group (*n* = 10 VDI-1/3/12 infants with 9 remaining at both 9 and 12 months and *n* = 10 VI-1/3/12 infants with 9 remaining at 12 months) and 10 unvaccinated Controls (*n* = 10 Control infants with 9 remaining at 12 months). Brown asterisks indicate future hospitalization with all-cause diarrhea and red asterisks indicate future hospitalization with *C. coli* diarrhea. Brown circles indicate animals that had been hospitalized with all-cause diarrhea whereas red circles indicate animals that were hospitalized with *C. coli*-associated diarrhea. Vaccinated infants born to vaccinated dams (VDI-1/3/12) were consistent in having the highest numerical average level of tryptophan in their serum at 1, 3, 6, 9, and 12 months of age, although these levels were only significantly higher than unvaccinated Controls at the 6 month time point (two-sided ANOVA Dunnett-adjusted P=0.02). In contrast, vaccinated infants born to unvaccinated dams (VI-1/3/12) were similar to unvaccinated Controls at each time point with no statistically significant differences noted (two-sided ANOVA Dunnett-adjusted P≥0.58). Interestingly, in these studies the largest difference in serum tryptophan levels among infant macaques at 6 months of age were preceded by the largest observed differences in growth trajectories between VDI-1/3/12 and Controls was identified at 9 months of age (Fig. 5). Source data are provided as a Source Data file.
