# Supplementary material for: Campylobacter vaccination reduces diarrheal disease and infant growth stunting among rhesus macaques
Source: Nat Commun. 2023 Jun 26;14:3806. doi: 10.1038/s41467-023-39433-1 (PMC10293212; doi:10.1038/s41467-023-39433-1)
Supplement: Supplementary file 3 — Reporting Summary [file 41467_2023_39433_MOESM3_ESM.pdf]

nature portfolio

Corresponding author(s): Mark K.Slifka

Last updated by author(s): 06-08-23

Reporting Summary

Nature Portfolio wishes to improve the reproducibility of the work that we publish. This form provides structure for consistency and transparency in reporting. For further information on Nature Portfolio policies, see our Editorial Policies and the Editorial Policy Checklist.

Statistics

For all statistical analyses, confirm that the following items are present in the figure legend, table legend, main text, or Methods section.

n/a	Confirmed	
☐	☒	The exact sample size (*n*) for each experimental group/condition, given as a discrete number and unit of measurement
☐	☒	A statement on whether measurements were taken from distinct samples or whether the same sample was measured repeatedly
☐	☒	The statistical test(s) used AND whether they are one- or two-sided<br>*Only common tests should be described solely by name; describe more complex techniques in the Methods section.*
☐	☒	A description of all covariates tested
☐	☒	A description of any assumptions or corrections, such as tests of normality and adjustment for multiple comparisons
☐	☒	A full description of the statistical parameters including central tendency (e.g. means) or other basic estimates (e.g. regression coefficient) AND variation (e.g. standard deviation) or associated estimates of uncertainty (e.g. confidence intervals)
☐	☒	For null hypothesis testing, the test statistic (e.g. *F*, *t*, *r*) with confidence intervals, effect sizes, degrees of freedom and *P* value noted<br>*Give P values as exact values whenever suitable.*
☒	☐	For Bayesian analysis, information on the choice of priors and Markov chain Monte Carlo settings
☒	☐	For hierarchical and complex designs, identification of the appropriate level for tests and full reporting of outcomes
☒	☐	Estimates of effect sizes (e.g. Cohen's *d*, Pearson's *r*), indicating how they were calculated

Our web collection on statistics for biologists contains articles on many of the points above.

Software and code

Policy information about availability of computer code

Data collection	SoftMax Pro 6.5.1, Illumina MiSeq Control Software v3.1.0, LabSolutions Software, V5.72
Data analysis	GraphPad Prism Version 9.3.1 (350) , QIIME2 analysis pipeline, DADA2 Pipeline, MAFFT , FastTree2, QIIME 2, ADONIS from the R package Vegan46 , R4.1.1 (R Foundation for Statistical Computing, Vienna, Austria, 2019), SAS9.4 (SAS Institute, Cary, NC)

For manuscripts utilizing custom algorithms or software that are central to the research but not yet described in published literature, software must be made available to editors and reviewers. We strongly encourage code deposition in a community repository (e.g. GitHub). See the Nature Portfolio guidelines for submitting code & software for further information.

Data

Policy information about availability of data

All manuscripts must include a data availability statement. This statement should provide the following information, where applicable:

- Accession codes, unique identifiers, or web links for publicly available datasets
- A description of any restrictions on data availability
- For clinical datasets or third party data, please ensure that the statement adheres to our policy

The 16s rRNA data generated in this study have been deposited in the Sequence Read Archive (SRA) database under accession code PRJNA896946 [https://www.ncbi.nlm.nih.gov/bioproject/PRJNA896946]. Source data are provided with this paper.

Human research participants

Policy information about studies involving human research participants and Sex and Gender in Research.

Reporting on sex and gender	N/A
Population characteristics	N/A
Recruitment	N/A
Ethics oversight	N/A

Note that full information on the approval of the study protocol must also be provided in the manuscript.

Field-specific reporting

Please select the one below that is the best fit for your research. If you are not sure, read the appropriate sections before making your selection.

☒ Life sciences ☐ Behavioural & social sciences ☐ Ecological, evolutionary & environmental sciences

For a reference copy of the document with all sections, see nature.com/documents/nr-reporting-summary-flat.pdf

Life sciences study design

All studies must disclose on these points even when the disclosure is negative.

Sample size	Sample size was determined/limited by availability of subjects/samples. Infant macaques were studied across two birth cohorts (2017 and 2018) to increase sample size.
Data exclusions	Rhesus macaque infants born in outdoor shelters from March through July in 2017 and 2018 were included in the analysis with the following exclusions: No animals were used from shelter groups that were involved with prior Campylobacter vaccination studies or undergoing treatment for alopecia.
Replication	16S amplicon sequencing were performed in replicates. In order to minimize batch effect, samples were selected at random and sequenced across multiple 16s rRNA sequencing runs. Negative controls collected during every DNA extraction were also included. A non-template control (Nuclease-free molecular grade water) and positive template control (Microbial Community DNA Standard, Zymo #D6306) were utilized during each 16s library preparation. Samples lacking sufficient sequencing depth were re-sequenced.<br>Samples were tested at least in duplicate for antibody measurement ELISAs and and paired samples with>25% coefficient of variation (CV) were repeated. All attempts at replication were successful.
Randomization	The subject allocations were not formally randomized but infant sex, weight, length, or BMI were not known at the time of selection to a particular study group. Rhesus macaques (Macacca mulatta) were housed in outdoor sheltered breeding groups consisting of approximately 20-50 animals per shelter and an average of 3 infants (range, 1-15) were vaccinated against Campylobacter per shelter group in 2017 and an average of 2 infants (range, 1-5) were vaccinated against Campylobacter per shelter group in 2018.
Blinding	Investigators were not blinded to the group allocation, but the veterinary care staff who monitored diarrheal incidence in the colony, technicians who performed quantitative K/T analysis, microbiological testing and ELISAs were blinded to the group allocation.

Reporting for specific materials, systems and methods

We require information from authors about some types of materials, experimental systems and methods used in many studies. Here, indicate whether each material, system or method listed is relevant to your study. If you are not sure if a list item applies to your research, read the appropriate section before selecting a response.

Materials & experimental systems

n/a	Involved in the study	
☐	☒	Antibodies
☒	☐	Eukaryotic cell lines
☒	☐	Palaeontology and archaeology
☐	☒	Animals and other organisms
☒	☐	Clinical data
☒	☐	Dual use research of concern

Methods

n/a	Involved in the study	
☒	☐	ChIP-seq
☒	☐	Flow cytometry
☒	☐	MRI-based neuroimaging

Antibodies

Antibodies used	Mouse Anti-Monkey IgG-HRP (SB108a) Cat. No.: 4700-05 Southern Biotech,<br>Goat Anti-Monkey IgA-HRP Cat. No: orb21435 Biorbyt Ltd
Validation	Mouse Anti-Monkey IgG-HRP (SB108a)Cat. No.: 4700-05 has been quality tested for ELISA and FLISA by the manufacturer. Working dilutions for ELISA ranges from 1:4,000 – 1:8,000. The anitbody is specific to Rhesus and cynomolgus IgG with inimal reactivity to human and rabbit immunoglobulins, goat IgG, and mouse, rat, hamster, guinea pig, sheep, donkey, bovine, horse, porcine, feline, and chicken serum<br><br>Goat Anti-Monkey IgA-HRP Cat. No: orb21435 - following description is provided by the manufactuer:<br>"Horseradish peroxidase-conjugated IgG fraction of polyclonal goat antiserum to Monkey IgA, Fc specific. In enzyme-immunocytochemical and immunohistochemical staining for the detection of IgA at the cellular and subcellular level by staining of appropriately treated cell and tissue substrates; to demon- strate circulating IgA antibodies in serodiagnostic microbiology and autoimmune diseases; to identify a specific antigen using a reference antibody of monkey origin known to be of the IgA isotype in the middle layer of the indirect test procedure; in non-isotopic assay methodology (e.g. ELISA) to measure IgA in monkey serum or other body fluids. Antisera to IgA do not discriminate between serum IgA (monomeric and dimeric) and higher molecular forms such as secretory IgA. This immunoconjugate is not pre-diluted. The optimum working dilution of each conjugate should be established by titration before being used. Excess labelled antibody must be avoided because it may cause high unspecific background staining and interfere with the specific signal. Working dilutions for histochemical and cytochemical use are usually between 1:100 and 1:500; in ELISA and comparable non-precipitating antibody-binding assays between 1:1,000 and 1:10,000."

Animals and other research organisms

Policy information about studies involving animals; ARRIVE guidelines recommended for reporting animal research, and Sex and Gender in Research

Laboratory animals	Rhesus macaques (Macacca mulatta) , Male/Female 1 month old to 18 years old
Wild animals	The study did not involve wild animals
Reporting on sex	Infants were enrolled in the study prior to knowing their sex since this requires sedation of the dam and physical examination of the infant. The vaccinated rhesus macaque infants in the study were 55% female and 45% male, indicating no overall bias in enrollment. Sex was a variable used for the analysis of linear growth patterns in terms of LAZ scores by the statistical model
Field-collected samples	The study involved outdoor-housed Rhesus macaques
Ethics oversight	All animal work was approved by the Oregon National Primate Research Center (ONPRC) Institutional Animal Care and Use Committee (IACUC protocol: IP00000416) and performed in strict accordance with the recommendations described in the Guide for the Care and Use of Laboratory Animals of the National Institute of Health, the Office of Animal Welfare and the United States Department of Agriculture.

Note that full information on the approval of the study protocol must also be provided in the manuscript.
